# The Use of D-Optimal Mixture Design in Optimizing Development of Okara Tablet Formulation as a Dietary Supplement

**DOI:** 10.1155/2015/684319

**Published:** 2015-06-11

**Authors:** Nur Izzati Mohamad Zen, Siti Salwa Abd Gani, Rosnah Shamsudin, Hamid Reza Fard Masoumi

**Affiliations:** ^1^Halal Product Research Institute, Universiti Putra Malaysia (UPM), 43400 Serdang, Selangor, Malaysia; ^2^Centre of Foundation Studies for Agriculture Science, Universiti Putra Malaysia (UPM), 43400 Serdang, Selangor, Malaysia; ^3^Department of Chemistry, Faculty of Science, Universiti Putra Malaysia (UPM), 43400 Serdang, Selangor, Malaysia; ^4^Department of Process and Food Engineering, Faculty of Engineering, Universiti Putra Malaysia (UPM), 43400 Serdang, Selangor, Malaysia

## Abstract

The usage of soy is increasing year by year. It increases the problem of financial crisis due to the limited sources of soybeans. Therefore, production of oral tablets containing the nutritious leftover of soymilk production, called okara, as the main ingredient was investigated. The okara tablets were produced using the direct compression method. The percentage of okara, guar gum, microcrystalline cellulose (Avicel PH-101), and maltodextrin influenced tablets' hardness and friability which are analyzed using a D-optimal mixture design. Composition of Avicel PH-101 had positive effects for both hardness and friability tests of the tablets. Maltodextrin and okara composition had a significant positive effect on tablets' hardness, but not on percentage of friability of tablets. However, guar gum had a negative effect on both physical tests. The optimum tablet formulation was obtained: 47.0% of okara, 2.0% of guar gum, 35.0% of Avicel PH-101, and 14.0% of maltodextrin.

## 1. Introduction

Okara is the by-product of the soybean milk and tofu production. The main component of okara is fiber (about 50%) which is composed of cellulose, hemicellulose, and lignin. Okara also contains about 25% protein, 10% oil, and low amounts of starch and simple carbohydrates. Okara is also known as low-cost nutrient rich fibers in soybean [1]. According to Bowles and Demiate [2], one-third of the total amount of isoflavones remains in okara. Isoflavones have many advantages for health such as acting as an antioxidant and preventing chronic diseases like cancer and heart disease [2]. Obesity is strongly related to other chronic diseases like cardiovascular diseases and diabetes [3]. One of the methods that can manage and prevent an obesity problem is the usage of fiber-rich foods due to providing high fiber food intake satiety [4]. Hence, okara could be very effective as a dietary supplement.

There are many forms of dietary supplements, for example, tablets, capsules, liquids, powders, and gels. Dietary supplements are different from drugs, and they are nonpotent drugs. The Food and Drug Administration (FDA) defined a dietary supplement as an alternative food containing essential nutrients like vitamins, minerals, and proteins. Subsequently, the Nutrition Labeling and Education Act of 1990 added “herb or nutritional substances” to the definition. In the pharmaceutical industry, tablets are the most acceptable form for consumers in comparison with other oral dosage forms [5]. Tablet oral dosage has many advantages such as its ease of handling, chemical and physical stability, and portability. Furthermore, this type of dosage form ensures accuracy and consistency of dosages [6]. There are many examinations that can be done in order to maintain the physical qualities of the tablets, for example, hardness test, percentage of friability test, disintegration test, and dissolution test [7].

Tablets are mixtures of active ingredients and other excipients. Mixtures mean the sum of all the ingredients is 100% [8]. There are many types of excipient with their own function in dosage formulation: diluents or fillers, binders, lubricants, glidants, antiadherents, disintegrants, colorants, and flavor or sweeteners. The mixture design statistical method is the most suitable method used in optimizing the tablet production process. The mixture design method is usually used in mixture formulation [9]. For example, in three components of formulation,

(1) where  i=1,2,30≤xi≤1,x1+x2+x3=1.

There are many types of mixture design: simplex-lattice design, simplex-centroid design, axial design, and D-optimal design. In this study, computed-generated D-optimal mixture design was used. D-optimal design is constructed to minimize the overall variance of the predicted regression coefficient by maximizing the value of determinant of the information matrix [10]. The benefits of D-optimal configuration, the experimental region is not simplex but it is irregular [11]. As compared with other designs, D-optimal design has a smaller number of runs and thus needs low cost of experimentation. Furthermore, combined mixture and process variables can be used in the same experimental design [8].

In this present work, okara and other excipients used in tablet production were optimized using D-optimal mixture design in order to meet the physical properties of the tablet in terms of hardness and friability.

## 2. Materials and Methods

### 2.1. Materials

#### 2.1.1. Freeze-Dried Okara

A sample of okara was taken from a soymilk vendor in Serdang, Selangor, Malaysia. Prior to freeze drying of the okara, it was prefrozen at −20°C. The okara was then freeze-dried at −105°C for 120 hours.

#### 2.1.2. Excipients

The excipients were maltodextrin (R&M, UK), silicon dioxide (Merck, Germany), guar gum (R&M, UK), and Avicel PH-101 microcrystalline cellulose (FMC International, Philadelphia, PA).

### 2.2. Methods

#### 2.2.1. Preliminary Study of Screening the Variables

Preliminary study on tablet formulation was done by investigating the ingredients in tablet. Four excipients were chosen for okara tablet formulation based on their function. Three of them and okara were used as variables in D-optimal mixture design as they may have effect on the responses. The ranges of variables were also studied by using D-optimal mixture design software.

#### 2.2.2. Preparation of Okara Tablets Using D-Optimal Mixture Design

Parameters ranges for D-optimal mixture design in preparing okara tablets were prepared using Design-Expert Version 7.0 software as shown in Table 1. Okara tablet contains okara and four excipients: maltodextrin, guar gum, Avicel PH-101, and silicon dioxide. Four independent factors were studied: amount of okara (*A*), amount of guar gum (*B*), amount of maltodextrin (*C*), and amount of Avicel PH-101 (*D*). Also, two responses were examined: hardness and percentage of friability. These four variables generated 20 formulations of okara tablet with different composition of each ingredient as shown in Table 2.

#### 2.2.3. Tableting Using Direct Compression Method

The composition of okara, guar gum, Avicel PH-101, and maltodextrin was prepared according to data prepared by Design-Expert Version 7.0 software, while the composition of silicon dioxide was constant and they were mixed using the Glas-Col Dry Powder Rocking Shaker (Glas-Col, LLC, Terre Haute, IN, USA). The tableting processes were done with compression using the Instron 5566 (Instron Corporation, Norwood, MA, USA) at 9.8 kilonewtons (kN) with a velocity of 0.01 mm/s.

#### 2.2.4. Physical Evaluation of Tablets

*(1) Hardness of Okara Tablets*. Hardness or crushing strength of the tablets was measured using the Instron 5566 (Instron Corporation, Norwood, MA, USA) in diametric compression with a velocity of 0.016 mm/s. Hardness was expressed in kilonewton (kN).

*(2) Friability of Okara Tablets*. Friability of the tablets was evaluated using the Friabilator DF-3 (Distek Incorporation, North Brunswick, NJ, USA) with 25 rpm for four minutes, which means 100 rotations. The friability of tablets is expressed in percentage [12]:

(2) $$\begin{array}{c} \text{Friability},\%=\frac{\text{initial weight}-\text{final weight}}{\text{initial weight}}\times 100. \end{array}$$

#### 2.2.5. Statistical Analysis

D-optimal mixture design was used to determine the optimum amount of ingredients used in okara tablet formulation toward the responses, which are hardness and friability of the tablet. The statistical parameters used in evaluating and selecting the best-fitted model are coefficient of determination (*R* ^2^), adjusted coefficient of determination (adjusted *R* ^2^), predicted coefficient of determination (predicted *R* ^2^), coefficient of variation (C.V.), standard deviation, predicted residual sum of squares (PRESS), lack-of-fit, and regression data (*p* value and *F* value). The statistical analysis also constructs an equation from the best-fitted model. From the equation, the positivity of the coefficient presents the positive contribution toward the response, and vice versa. Also, a contour plot and three-dimensional response surface graph for each response were generated by Design-Expert Software Version 7.0 for a better explanation.

## 3. Results 

### 3.1. Screening the Range of Variables

Okara, maltodextrin, Avicel PH-101, and guar gum were chosen to be the variables toward the responses: hardness and friability. The levels of okara (25–50% w/w), maltodextrin (5–15% w/w), Avicel PH-101 (18–68% w/w), and guar gum (0–20% w/w) were selected (Table 1).

### 3.2. Hardness and Friability of Okara Tablets

Table 2 shows that there are 20 formulations in this physical tests analysis. The actual and predicted data sets of the physical tests were tabulated in Table 3.

#### 3.2.1. Effect of Variables to the Hardness of Tablets

**Figure 1:**
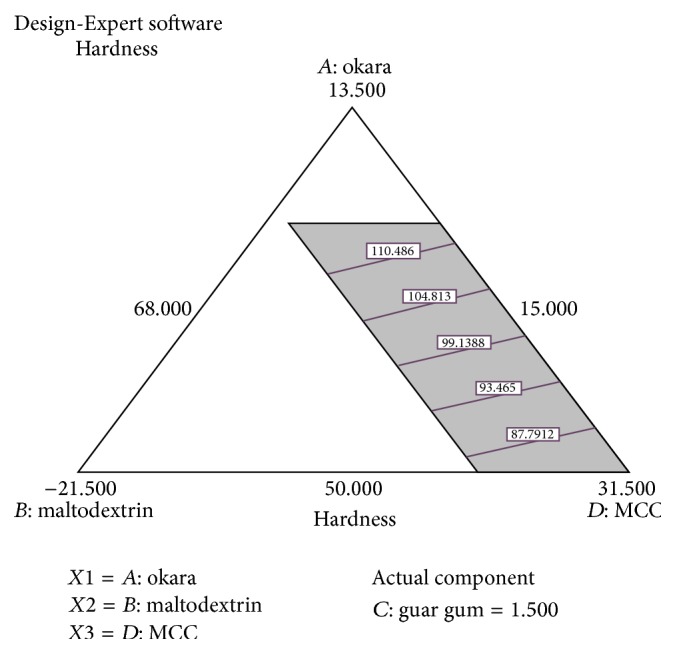
Contour diagram of relationship between three variables, (*A*) percentage of okara, (*B*) percentage of maltodextrin, and (*D*) percentage of Avicel PH-101, and with actual component of guar gum (*C* = 1.50%) to the hardness of the tablet.

**Figure 2:**
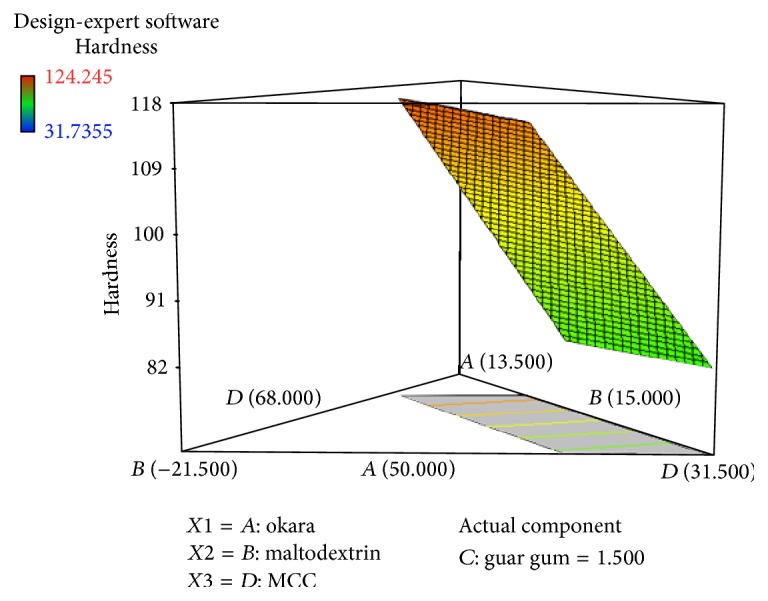
3D diagram of relationship between three variables, (*A*) percentage of okara, (*B*) percentage of maltodextrin, and (*D*) percentage of Avicel PH-101, and with actual component of guar gum (*C* = 1.50%) to the hardness of the tablet.

Figures 1 and 2 show the relationship between the hardness of a tablet and the deployed component variables (percentage of okara, maltodextrin, guar gum, and Avicel PH-101). The statistical analysis from Design-Expert Version 7.0 software suggested a linear model as the best model with *p* < 0.0001. The model is significant and the lack-of-fit is insignificant (Table 4). This means that the possibility of error occurring is low.

Final equation for hardness of okara tablet is

(3) Hardness,Y1=0.33327A+1.18672B−1.14021C+1.56709D.

#### 3.2.2. Effect of Variables on the Friability of Tablets

**Figure 3:**
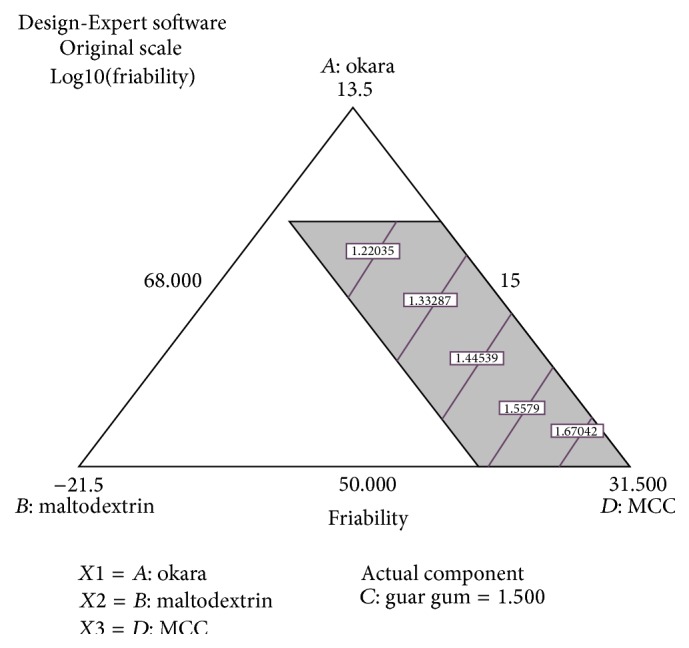
Contour diagram of relationship between three variables, (*A*) percentage of okara, (*B*) percentage of maltodextrin, and (*D*) percentage of Avicel PH-101, and with actual component of guar gum (*C* = 1.50%) to the friability of the tablet.

**Figure 4:**
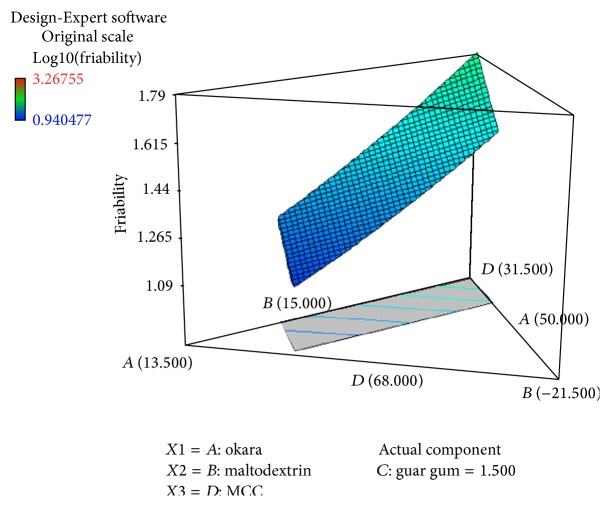
3D diagram of relationship between three variables, (*A*) percentage of okara, (*B*) percentage of maltodextrin, and (*D*) percentage of Avicel PH-101, and with actual component of guar gum (*C* = 1.50%) to the friability of the tablet.

Figures 3 and 4 show the relationship between the percentage of component variables and the friability of the tablet. The statistical analysis suggested a Log10 linear model with *p* < 0.0001. The model was significant and lack-of-fit was insignificant, which means the model demonstrates the goodness of fit (Table 4).

Final equation for friability of okara tablet is

(4) Log⁡10Friability,Y2=0.004255A+0.0047081B+0.013067C−0.0016455D.

### 3.3. Optimization Using D-Optimal Mixture Design

Design-Expert software generates 20 formulations to produce optimized okara tablet with acceptable hardness and friability. The optimum percentages of ingredients selected were as follows: okara 47.0%, maltodextrin 14.0%, guar gum 2.0%, and Avicel PH-101 35.0%. The recommended formulation was carried out and the actual values and predicted values of okara tablet hardness and percentage of friability were compared (Table 5).

## 4. Discussion

### 4.1. Screening of Variables

Preliminary studies were carried out to screen out the suitable variables for the responses and to select the ranges of variables in the okara tablet formulation. Initially, the excipients used in the okara tablet formulation were studied. Maltodextrin is selected as the binder, Avicel PH-101 was chosen as the filler, the guar gum was chosen as disintegrant, also, okara additionally tested as variables to study its impact toward reactions. For safety, the hardness should be more than 40 kN and the friability should be less than 2% [13]. The hardness and friability fall in the accepted ranges when the level of okara (25–50% w/w), maltodextrin (5–15% w/w), Avicel PH-101 (18–68% w/w), and guar gum (0–2% w/w).

### 4.2. Hardness and Friability of Okara Tablets

#### 4.2.1. Effect of Variables on the Hardness of Tablets

Figures 1 and 2 and (3) from hardness test show that a high Avicel PH-101 percentage gives the highest hardness because Avicel PH-101 is known as a filler-binder in tablets [14]. During compression, Avicel PH-101 which is made of fiber particles may undergo a process called fragmentation, which means the breakage of particles into the smaller units. The fragmentation process increases secondary binding points; consequently, tablet hardness gets stronger [15]. Avicel PH-101 also improves the flowability, compactibility, and compressibility of the tablets [15, 16]. The most important characteristic that the tablet filler should have is the compactibility. Strong tablets are made with densely compacted powder. Avicel PH-101 has the smallest particle size among other components, which is 50 *μ*m. Therefore, a smaller powder particle size will be more compactible compared to a larger powder particle size [17]. In addition, increasing the amount of maltodextrin increases the hardness of the tablet. Final equation for hardness of okara tablet and Figures 1 and 2 did prove that increasing the percentage of maltodextrin affects the hardness of an okara tablet. Maltodextrin, which is usually used as binder in tableting, has also a large effect on the hardness of the tablet. Maltodextrin can absorb and desorb moisture easily from the humidity due to its natural behavior as an amorphous sugar [18]. That explains how the binder provides cohesiveness within the powders in tablet formulation [19]. The composition of okara just slightly affects the hardness of the tablet and guar gum had a negative effect on hardness which is shown in (3). The main component of okara is fiber; hence, a larger percentage of okara can also exhibit greater hardness of the tablet. In conclusion, the hardness of the tablet is affected by the behavior of the powder. Thus, in the tablet formulation, Avicel PH-101 had the highest effect on tablet hardness due to its compactibility.

#### 4.2.2. Effect of Variables on the Friability of Tablets

Linear regression model of friability test generated adequate model with coefficient of determination (*R* ^2^ = 0.8807), but it can be improved. Sometimes due to inhomogeneity of variance and abnormality of errors in the model, transformation of model is an effective method to improve the ANOVA of friability test [20]. The Box-Cox plot from Design-Expert recommends natural log transformation (*λ* = 0) of the model. The advantages of model transformation are to stabilize the variance of residual and normalize the distribution of the residual. The final coefficient of determination (*R* ^2^ = 0.9103) is greater than the previous one. This result shows that the error has been corrected during the transformation. Figures 3 and 4 and (4) for friability test show that the Avicel PH-101 had a positive effect on the percentage of friability. Increasing the percentage of Avicel PH-101 in the tablets decreases the percentage of friability. According to Bastos et al. [21], microcrystalline cellulose improves both crushing strength and percentage of friability [21]. Segale et al. [22] reported that a fast melting tablet (FMT) containing Avicel PH-101 exhibits low friability compared to maltodextrin. FMT containing maltodextrin is weaker than FMT containing Avicel PH-101 [22]. The final equation of friability also showed that maltodextrin does have an effect on the percentage of friability. Also, okara has a slightly negative effect on maintaining tablet hardness. Guar gum is not suitable to be a tablet binder since increasing guar gum makes the tablet more friable. Thus, the percentage of guar gum should be low to ensure the good physical quality of tablets. Therefore, a high percentage of Avicel PH-101 in tablet formulation can keep the tablet from breaking easily.

### 4.3. Verification of the Model

In order to verify the optimum formulation generated from the Design-Expert software, verification of data is needed. The results were verified by relative standard error (RSE):

(5) $$\begin{array}{c} \text{RSE},\%=\frac{\text{Actual value}-\text{Predicted value}}{\text{Predicted value}}\times 100. \end{array}$$

The optimized formulation of okara tablet (Table 5) showed nonsignificant difference between the predicted values and actual values for both hardness and percentage of friability of tablet. Therefore, the finalized equation for hardness and percentage of friability generated by Design-Expert 7.0 software is acceptable to be used in okara tablet formulation.

## 5. Conclusion

Hardness of the tablets cannot be evaluated by individual usage of the crushing strength test; but the friability test is also necessary. Avicel PH-101 showed a positive effect for both physical tests: hardness and friability. Therefore, the percentage of Avicel PH-101 should be large to make the tablet stronger. The percentage of maltodextrin can be at the medium level. The percentage of okara as the principle ingredient ought to be as high as not influencing the physical properties. They had a positive effect on tablet hardness, but not on the percentage of friability. Guar gum has no positive effect on both physical tests but is needed as the disintegrant of the tablet. Therefore, it is limited to a low amount of tablet formulation.

## Figures and Tables

**Table 1:** Parameters studied in physical optimization of okara tablet.

Code	Parameters	Low level	High level
*A*	Percentage of okara	25	50
*B*	Percentage of maltodextrin	5	15
*C*	Percentage of guar gum	0	20
*D*	Percentage of Avicel PH-101	18	68

**Table 2:** Percentage composition of okara tablet (100%).

Ingredients	*A*	*B*	*C*	*D*	Silicon dioxide
F1	25.05	15.00	0.00	57.95	2.000
F2	49.97	15.00	0.81	32.21	2.000
F3	50.00	5.39	7.05	35.56	2.000
F4	25.02	5.00	0.00	67.98	2.000
F5	25.00	8.94	15.22	48.84	2.000
F6	38.02	5.34	20.00	34.64	2.000
F7	40.21	9.20	0.00	48.58	2.000
F8	50.00	9.86	19.98	18.16	2.000
F9	26.01	14.99	20.00	37.00	2.000
F10	36.95	15.00	10.50	35.55	2.000
F11	36.53	5.00	11.57	44.91	2.000
F12	26.73	5.14	7.88	58.26	2.000
F13	45.52	11.65	13.27	27.55	2.000
F14	30.61	15.00	5.76	46.63	2.000
F15	43.06	11.60	3.80	39.55	2.000
F16	49.97	15.00	0.81	32.21	2.000
F17	50.00	5.39	7.05	35.56	2.000
F18	26.01	14.99	20.00	37.00	2.000
F19	25.05	15.00	0.00	57.95	2.000
F20	25.02	5.00	0.00	67.98	2.000

**Table 3:** Actual and predicted values for hardness and friability of okara tablet formulation.

Ingredients	Actual hardness (kN)	Predicted hardness (kN)	Hardness RSE (%)	Actual friability (%)	Predicted friability (%)	Friability RSE (%)
F1	116.84	116.96	0.1017	1.342	1.147	16.9891
F2	83.78	84.01	0.2697	1.731	1.730	0.0602
F3	68.37	70.75	3.3625	1.896	1.957	3.1653
F4	124.25	120.81	2.8433	0.940	0.968	2.7952
F5	73.88	78.13	5.4446	1.229	2.012	38.8902
F6	48.45	50.49	4.0368	2.382	2.53023	5.8732
F7	83.73	100.46	16.6572	1.236	1.366	9.5770
F8	39.16	34.03	15.0890	3.268	2.865	14.0424
F9	60.25	61.64	2.2494	2.600	2.447	6.2418
F10	71.84	73.85	2.7251	1.670	2.073	19.4233
F11	46.28	75.28	38.5186	2.179	1.953	11.5650
F12	84.65	97.32	13.0164	1.310	1.511	13.2689
F13	31.74	57.04	44.3645	2.044	2.373	13.8413
F14	97.88	94.50	3.5751	1.671	1.635	2.1961
F15	81.81	85.76	4.6016	1.755	1.713	2.4478
F16	83.78	84.01	0.2697	1.878	1.730	8.5422
F17	72.52	70.75	2.5033	1.881	1.957	3.9171
F18	64.24	61.64	4.2255	2.295	2.447	6.2346
F19	116.84	116.96	0.1017	1.229	1.147	7.0760
F20	121.21	120.81	0.3319	1.064	0.968	10.0074

**Table 4:** Data from analysis of variance (ANOVA) for hardness and friability of okara tablet.

	Hardness	Friability
Model	Significant	Significant
*R* ^2^	0.9893	0.9103
Adjusted *R* ^2^	0.9866	0.8911
Predicted *R* ^2^	0.9810	0.8635
*p* value	0.1243	0.1047
*F* value	2.97	3.23
C.V. %	3.64	19.96
Lack-of-fit	Not significant	Not significant
Standard deviation	3.020	0.046
PRESS	193.390	0.044

Note: *p* < 0.05 is significant.

*p* > 0.05 is not significant.

**Table 5:** Predicted and actual values of hardness and percentage of friability of optimal formulation.

Independent variables				Hardness			Friability		
*A* (%)	*B* (%)	*C* (%)	*D* (%)	Actual value	Predicted value	RSE (%)	Actual value	Predicted value	RSE (%)
47	14	2	35	84.922	84.845	0.0908	1.697	1.714	0.9918
